# The Landscape of Severe Combined Immunodeficiency Newborn Screening in the United States in 2020: A Review of Screening Methodologies and Targets, Communication Pathways, and Long-Term Follow-Up Practices

**DOI:** 10.3389/fimmu.2020.577853

**Published:** 2020-10-28

**Authors:** Ruthanne Sheller, Jelili Ojodu, Emma Griffin, Sari Edelman, Careema Yusuf, Trey Pigg, Alissa Huston, Brian Fitzek, John G. Boyle, Sikha Singh

**Affiliations:** ^1^ Newborn Screening and Genetics Department, Association of Public Health Laboratories, Silver Spring, MD, United States; ^2^ Immune Deficiency Foundation, Towson, MD, United States

**Keywords:** severe combined immunodeficiency, newborn screening (NBS), United States, communication pathways, long-term follow-up, education

## Abstract

Severe combined immunodeficiency (SCID) is T cell development disorders in the immune system and can be detected at birth. As of December 2018, all 53 newborn screening (NBS) programs within the United States and associated territories offer universal screening for SCID. The Association of Public Health Laboratories (APHL), along with the Immune Deficiency Foundation (IDF), surveyed public health NBS system laboratory and follow-up coordinators regarding their NBS program’s screening methodologies and targets, protocols for stakeholder notifications, and long-term follow-up practices. This report explores the variation that exists across NBS practices, revealing needs for efficiencies and educational resources across the NBS system to ensure the best outcomes for newborns.

## Introduction

In the United States, newborn screening (NBS) is a state public health service designed to identify individuals in a population at high risk of certain congenital or heritable disorders. With approximately four million newborns screened for at least 30 primary conditions every year, NBS is recognized as one of the most successful public health programs in the US (1).

While the Health and Human Services (HHS) Federal Advisory Committee on Heritable Disorders in Newborns and Children (ACHDNC) recommends disorders to be included on the Recommended Uniform Screening Panel (RUSP), each state determines the specific disorders for which it screens. In the United States, there are significant variations in geographic, population, and demographic characteristics within and between states. As such, each state NBS system applies independent criteria to determine the timeline with which they will implement NBS for newly added RUSP conditions (2).

In May of 2010, severe combined immunodeficiency (SCID) was added to the RUSP. SCID are T-cell developmental disorders in the immune system, affecting approximately one in 58,000 births in the United States (3). Screening also identifies one in 20,000 newborns who have non-SCID T-cell lymphopenia (TCL) (4). Newborns with SCID (and TCL) appear healthy in the neonatal period, but are extremely vulnerable to infection. Exposure to common infections and live vaccines is life threatening unless timely treatment is provided in infancy. NBS contributes to optimized health outcomes for newborns with SCID through earlier detection, diagnosis, and treatment (5).

The addition of SCID to NBS panels posed both opportunities and challenges to NBS programs, requiring programs to integrate new screening technology within their laboratories, to train NBS personnel on this technology, and to establish clinical referral networks (6). As of December 2018, all 53 NBS programs within the United States and associated territories offer SCID NBS through the utilization of dried blood spot cards (**Supplementary Document**) (4, 7).

While the primary targets of SCID NBS are typical SCID, leaky SCID, and Omenn syndrome, additional conditions with low or absent T-cell numbers have been detected as secondary targets (5). NBS programs continue to partner with clinicians and the Newborn Screening Technical assistance and Evaluation Program (NewSTEPs) to collect SCID case-level data categorized by public health surveillance case definitions. These surveillance case definitions allow for consistent public health diagnoses and provide an estimate of the true birth prevalence of disorders identified by NBS (8).

In order to explore the variation that exists across SCID NBS practices in the US, the Association of Public Health Laboratories (APHL), in partnership with the Immune Deficiency Foundation (IDF), developed and fielded a survey to 53 NBS programs. The results revealed areas for addressing communication and follow-up inefficiencies as well as educational needs.

## Materials and Methods

In 2018, the IDF began a collaboration with APHL to implement the SCID Compass Program (9). Specifically, APHL provides technical assistance, trainings and resources to support SCID NBS. A component of the SCID Compass Program is to gain a clearer understanding of the disparate landscape of SCID NBS across all states and territories in the US. In doing so, APHL and IDF developed a web-based survey instrument using the Qualtrics software platform consisting of yes/no, open-ended, and multiple choice questions (**Supplementary Document**). This survey assessed NBS programs’ laboratory methodologies and targets, protocols for stakeholder notifications, and long-term follow-up practices as they pertain to SCID.

The survey was distributed to laboratory and follow-up coordinators in all 50 states, as well as the District of Columbia, Guam, and Puerto Rico (N = 53). Survey respondents were encouraged to reach out to their respective laboratory or follow-up counterparts to submit collaborative responses, and were requested to complete one survey per state.

Over the course of 6 weeks, APHL staff followed-up with NBS programs *via* email and phone with reminders. Surveys were submitted electronically through the Qualtrics software platform. The following software programs supported subsequent survey data analysis efforts: Microsoft Excel, Microsoft Power BI, and Python.

Supplementary data was also received from the NewSTEPs data repository. NewSTEPs is a national NBS resource center designed to provide data, technical assistance and training to NBS programs and assist states with quality improvement initiatives. NewSTEPs provides a centralized and secure online national data repository designed to collect comprehensive data on US NBS programs, inclusive of state profile data, confirmed cases and quality indicators. State profile data, including the screening methodologies and targets used in this report, is public facing. Most data elements are summarized and reported in real time on the NewSTEPs website and data repository in the form of interactive Tableau maps, tables and reports (1).

The survey had four sections: 1) Screening Methodologies and Targets, 2) Communication Pathways, 3) Education, and 4) Long-Term Follow-Up. The results from each section are described below.

## Results

APHL received 50 completed surveys (n = 50/53; 94% response rate) from laboratory and follow-up coordinators, representing 49 states and 1 US territory over the course of 6 weeks.

### Screening Methodologies and Targets

For the screening methodologies and targets section of the survey (**Supplementary Document**), respondents were requested to review their NBS program’s information that had been submitted to the NewSTEPs Data Repository and indicate if any edits or updates were needed. Respondents who selected “yes” to edits needed were asked to identify their screening methods and targets for first- as well as for second-tier screening.

For first-tier screening for SCID, all programs use T-cell receptor excision circle (TREC) as a screening marker; 90% of NBS programs (n = 45/50) opted to implement SCID NBS using a laboratory-developed real-time polymerase chain reaction (PCR) method. Ten percent of the NBS programs (n = 5/50) elected to use a U.S. Food and Drug Administration (FDA)-approved end-point PCR method, the PerkinElmer EnLite Neonatal TREC Kit, which received approval in December 2014 (10).

Twenty percent of NBS programs (n = 10/50) reported performing second-tier screening for SCID. All ten of these programs reported using TREC as their marker for second-tier screening. One of these NBS programs contracts with an external laboratory to perform second-tier screening. One other NBS program noted that their second-tier screening involved retesting specimens using their first-tier method in triplicate.

NBS programs were then asked if they multiplex SCID NBS with Spinal Muscular Atrophy (SMA) screening, both of which could employ the same molecular technique. At the time, this survey was issued, 18 NBS programs were offering universal SMA NBS. Of the 18 NBS programs offering SMA screening, 67% of these NBS programs (n = 12/18) reported multiplexing with SMA.

### Communication Pathways

Respondents were asked to identify who (primary care physician (PCP)/other provider, immunology consultant, public health nurse, family, hospital or other submitter, other) they inform about an out-of-range newborn screen for SCID, as well as the method used to contact those stakeholders (telephone, letter, certified letter, fax, email, text, other). Respondents were able to select all of the responses that applied for questions under this section.

**Figure 1:**
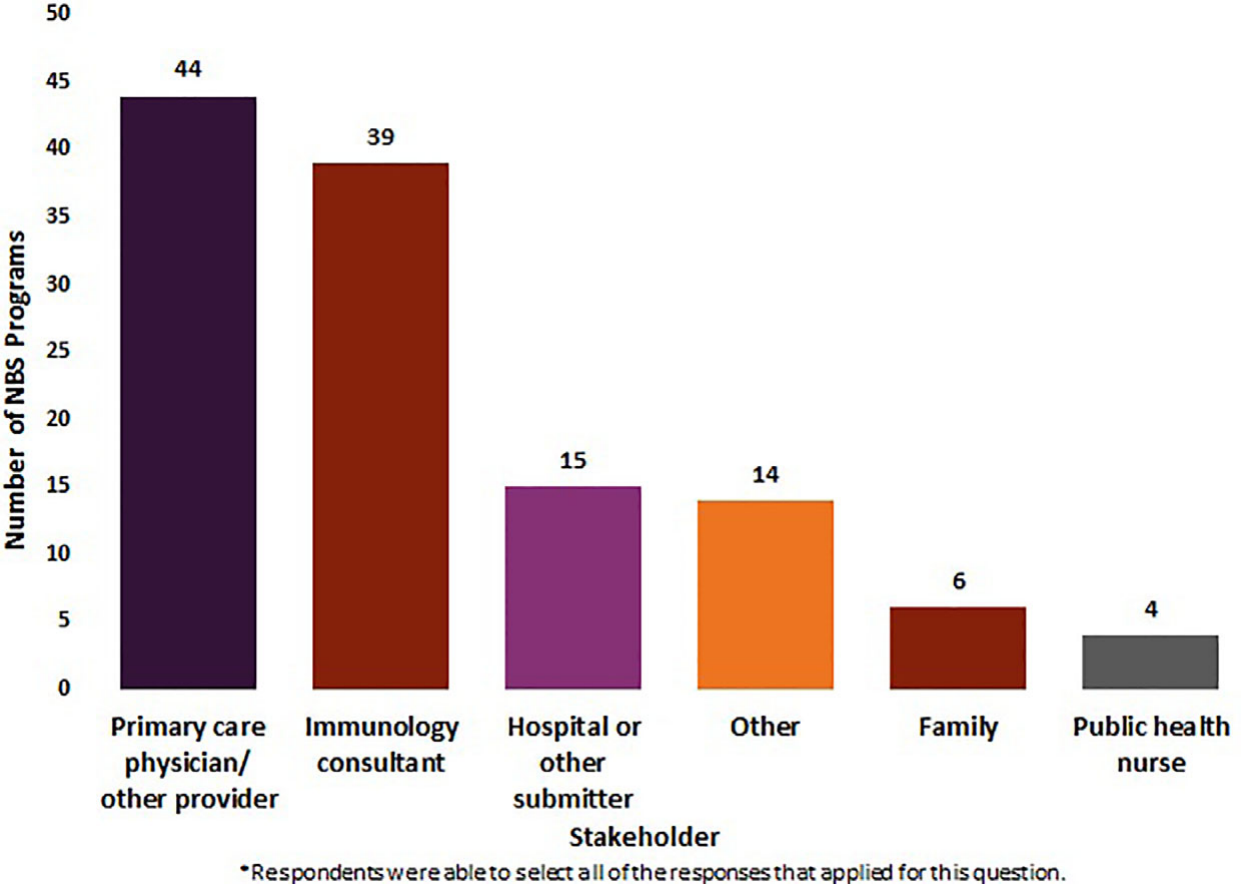
Stakeholders notified by newborn screening programs about an out-of-range screen for SCID (January 2020) (N = 50).

Fifty NBS programs (N = 50) reported on the stakeholders they notify about out-of-range screens for SCID. Eighty-eight percent (n = 44/50) of these NBS programs reported notifying PCP s/other providers of out-of-range screens. Seventy-eight percent (n = 39/50) notified immunology consultants, 30% (n = 15/50) notified hospitals or other submitters, 12% (n = 6/50) notified family, and 8% (n = 4/50) notified public health nurses. Twenty-eight percent of NBS programs (n = 14/50) indicated **“**other,**”** listing genetic coordinators, genetic referral centers, department of health coordinators, and midwives as other stakeholders that may be notified of an out-of-range SCID NBS screen. Twelve percent of NBS programs (n = 6/50) selected family, clarifying that families were only contacted if they were unable to get in touch with a PCP/other provider (**Figure 1**).

**Figure 2:**
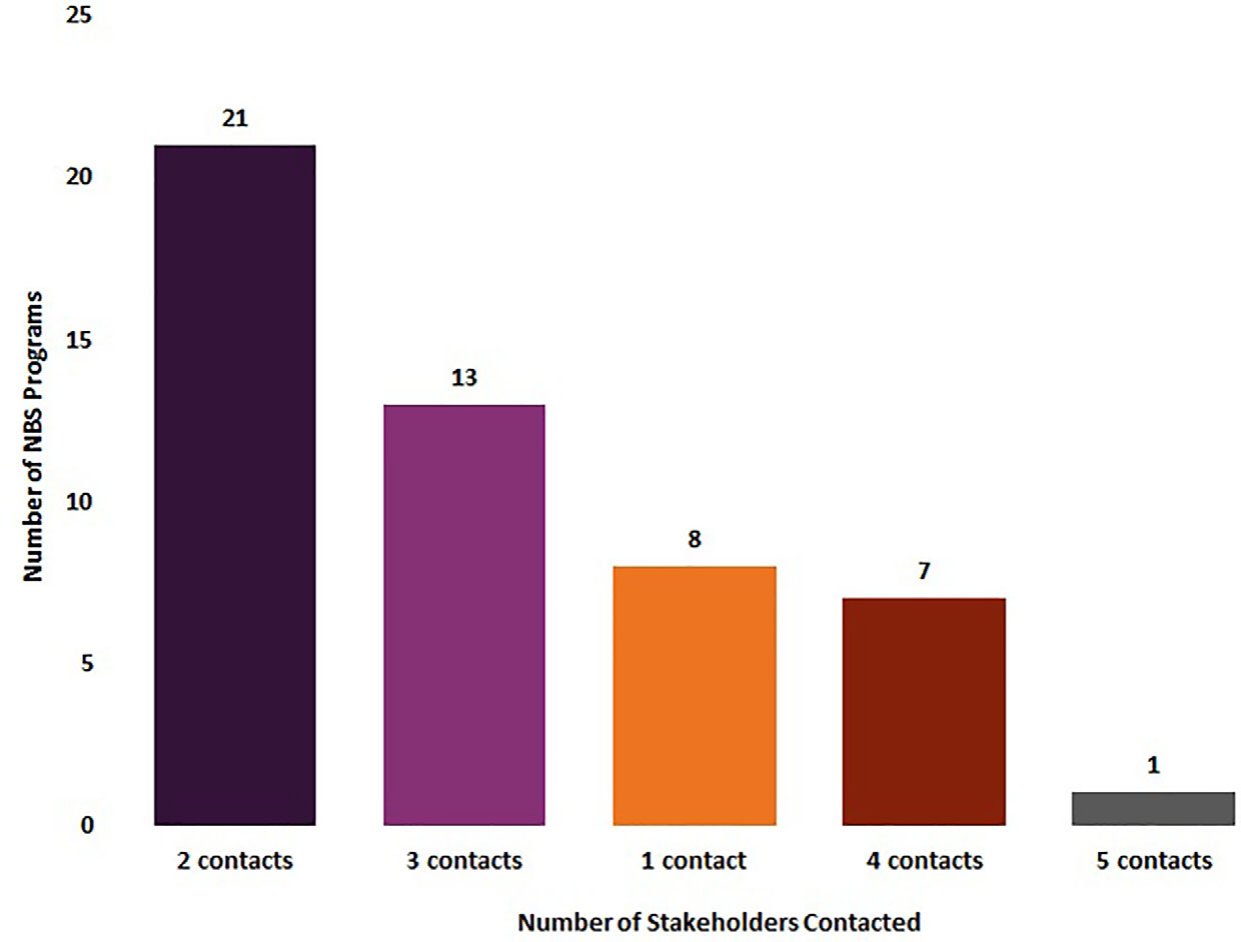
Number of stakeholders notified by newborn screening programs about an out-of-range screen for SCID (January 2020) (N = 50).

Eighty-four percent of NBS programs (n = 42/50) reported that they notified at least two groups of stakeholders of out-of-range SCID newborn screens, with 42% (n = 21/50) notifying two contacts, 26% (n = 13/50) notifying three contacts, 14% (n = 7/50) notifying four contacts, and 2% (n = 1/50) notifying five contacts. Sixteen percent of NBS programs (n = 8/50) only notified one stakeholder about an out-of-range SCID newborn screen (**Figure 2**).

**Figure 3:**
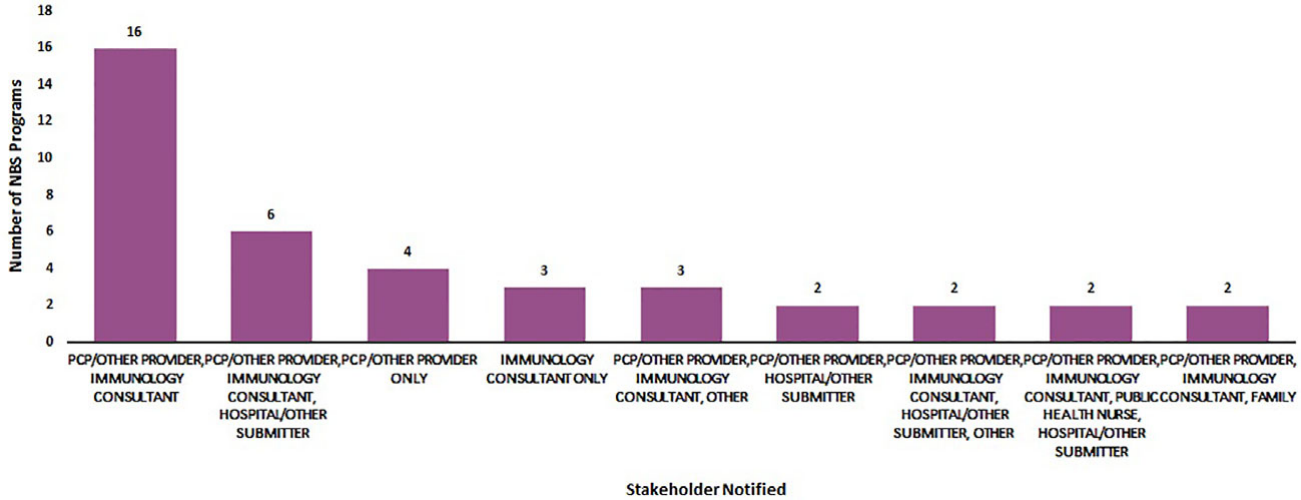
Most commonly notified stakeholders about an out-of-range newborn screen for SCID (January 2020) (N = 50).

**Figure 3** displays the various combinations of stakeholders that were notified of out-of-range screens. Primary care providers and immunologists were the most commonly notified stakeholders (n =16). Primary care providers, immunology consultants and hospitals/submitters were the second group of stakeholders most commonly notified (n = 6).

One-hundred percent of NBS programs (N = 50) reported the methods they utilize to notify stakeholders of an out-of-range SCID NBS screen. Ninety-eight percent of NBS programs (n = 49/50) utilized phones, 76% (n = 38/50) sent faxes, 40% (n = 20/50) email, 32% letter (n = 16/50), and 4% certified letter (n = 2/50). Three NBS programs (n = 3/50) noted the utilization of secure electronic systems to share results under “other”.

Forty percent of NBS programs (n = 20/50) reported they utilized three different contact methods to notify stakeholders of out-of-range SCID NBS screens. Thirty percent of NBS programs (n = 15/50) utilized two contact methods, 14% (n = 7/50) NBS programs utilized either one contact method or four contact methods, and 2% (1/50) utilized five contact methods.

**Figure 4:**
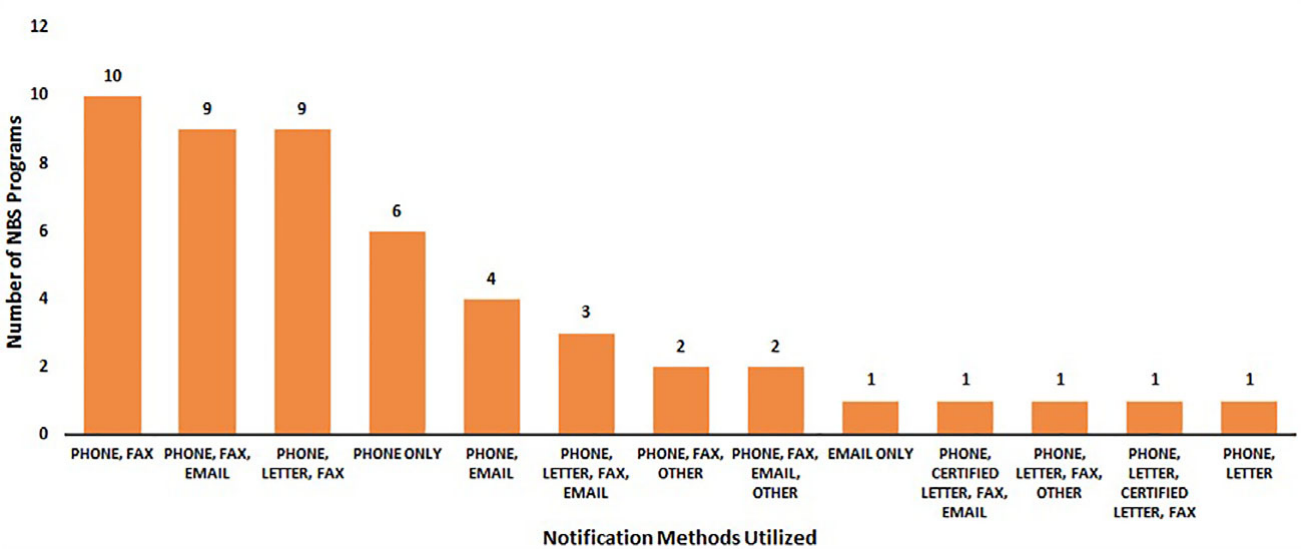
Most common notification methods regarding an out-of-range newborn screen for SCID (January 2020) (N = 50).

**Figure 4** displays the various combinations of contact methods that were utilized to notify stakeholders of out-of-range SCID NBS screens. Phone and fax (n = 10) were the most commonly used notification methods, followed by phone, fax, and email (n = 9) and phone, letter, and fax (n = 9).

### Education

Respondents were asked if they had a targeted education plan for health care providers, parents, and/or for the general public. Sixty-six percent of NBS programs (n = 33/50) reported they had a targeted education plan for health care providers. Sixty-three percent of NBS programs (n = 31/49) indicated they had a targeted education plan for parents and 23% (n = 10/43) reported that they had a targeted education plan for the general public. Twenty-one percent of NBS programs (n = 9/43) reported they maintained educational plans for all three stakeholder groups.

### Long-Term Follow-Up

Fifty-four percent of NBS programs (n = 27/50) reported that they do not follow patients after they have received a confirmed SCID diagnosis, with 4% of these NBS programs (n = 2/50) noting that they plan to initiate this practice in the coming year. Sixteen percent of NBS programs (n = 8/50) reported that they follow patients after they have received a confirmed diagnosis, from an age range of one year through 21 years (median = 10 years). Thirty-percent of NBS programs (n = 15/50) selected “other” as a response, with many NBS programs noting that once a patient receives a diagnosis, the patient is then followed by an immunologist, genetic specialist, clinical care center, social workers, or public health program such as the Children and Youth with Special Healthcare Needs Program within their state.

**Figure 5:**
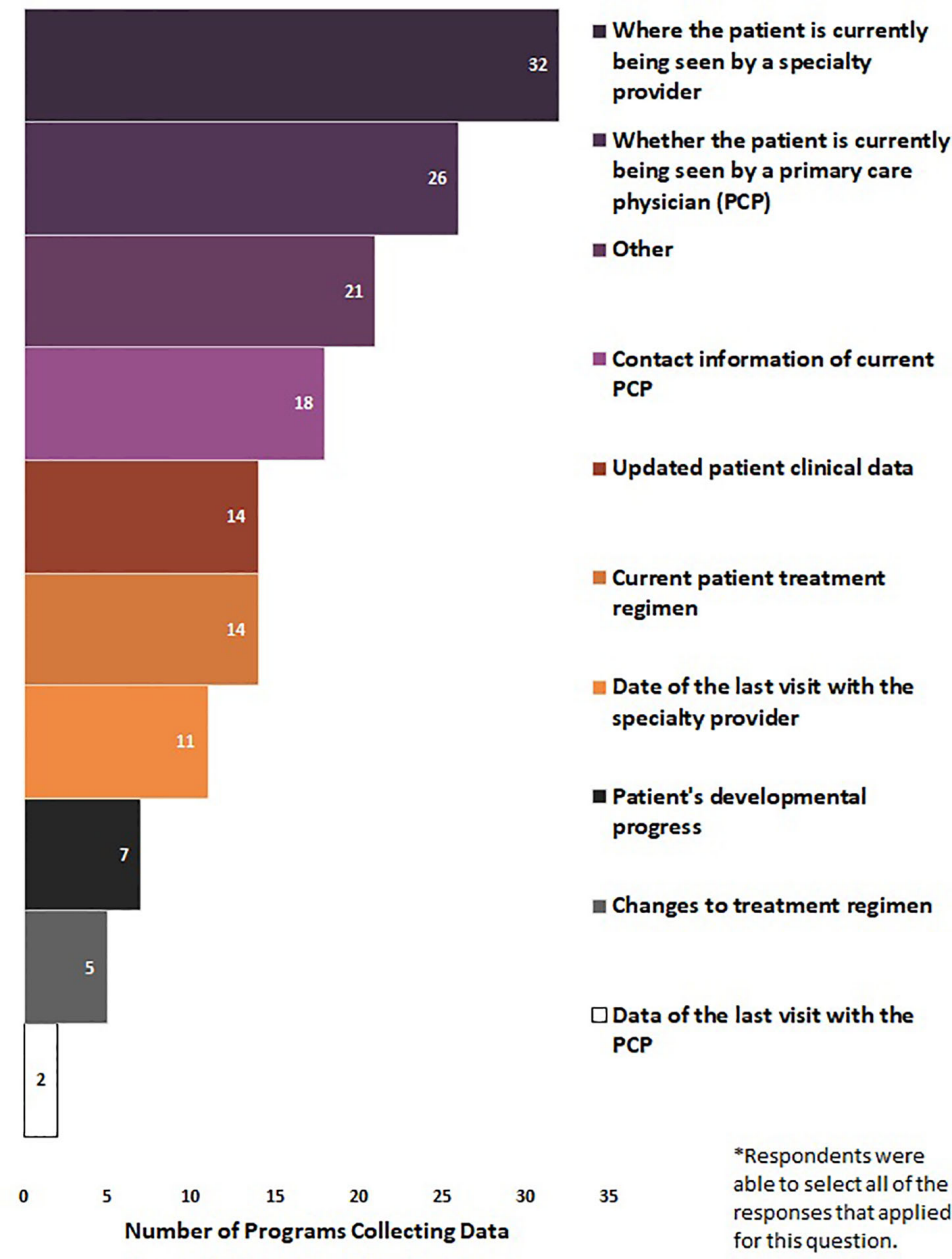
Long-term follow-up data elements collected by newborn screening programs for SCID (January 2020) (N = 41).

Eighty-two percent of NBS programs (N = 41/50) reported the elements for which they collected data on from patients who received an out-of-range SCID screen. Respondents were able to select all of the responses that applied for question. Seventy-eight percent of NBS programs (n = 32/41) collected where the patient is currently being seen by a specialty provider, 63% (n = 26/41) collected whether the patient is currently being seen by a PCP, 44% (n = 18/41) collected contact information of current PCP, 34% (n = 14/41) collected updated patient clinical data, 34% (n = 14/41) collected current patient treatment regimen, 27% (n = 11/41) collected date of last visit with the specialty provider, 17% (n = 7/41) collected patient’s developmental progress, 12% (n = 5/41) collected changes to treatment regimen, and 5% (n = 2/41) collected data of the last visit with the PCP. Fifty-one percent of NBS programs selected “other” (n = 21/41), noting additional data elements that were collected such as treatments and interventions (n = 2), informal updates (n = 3), specialist referral follow-up (n = 5), and clinical data and test results (n = 9) (**Figure 5**).

Sixty-six percent of NBS programs (N = 33/50) reported how collected data on patients who received abnormal SCID screens was utilized. Respondents were able to select all of the responses that applied for question. Seventy percent of NBS programs (n = 23/33) reported data was used to track the number of patients lost to follow-up. Fifty-eight percent of NBS programs (n = 19/33) indicated that data was used to track the clinical outcomes of patients. Thirty-three percent of NBS programs (n = 11/33) used data to assess the needs of patients/families for services. Twenty-seven percent of NBS Programs (n = 9/33) utilized data collected to evaluate the performance of specialty providers (physicians, nurses, and allied health professionals). Six percent of NBS programs (n = 2/33) used collected data to conduct research, such as cost-benefit analysis of screening. NBS programs also reported that data was used to ensure timely follow-up and treatment initiation, track the number of out-of-range, inconclusive, false positive, and false negative screens and associated diagnoses, and for broader publication reporting (i.e., Healthy People 2020) (11).

Seventy percent of NBS programs (n = 33/47) stated that the data elements listed above were entered into an electronic system or database. Thirty percent of NBS programs (n = 14/47) selected “no” in response to this question.

**Figure 6:**
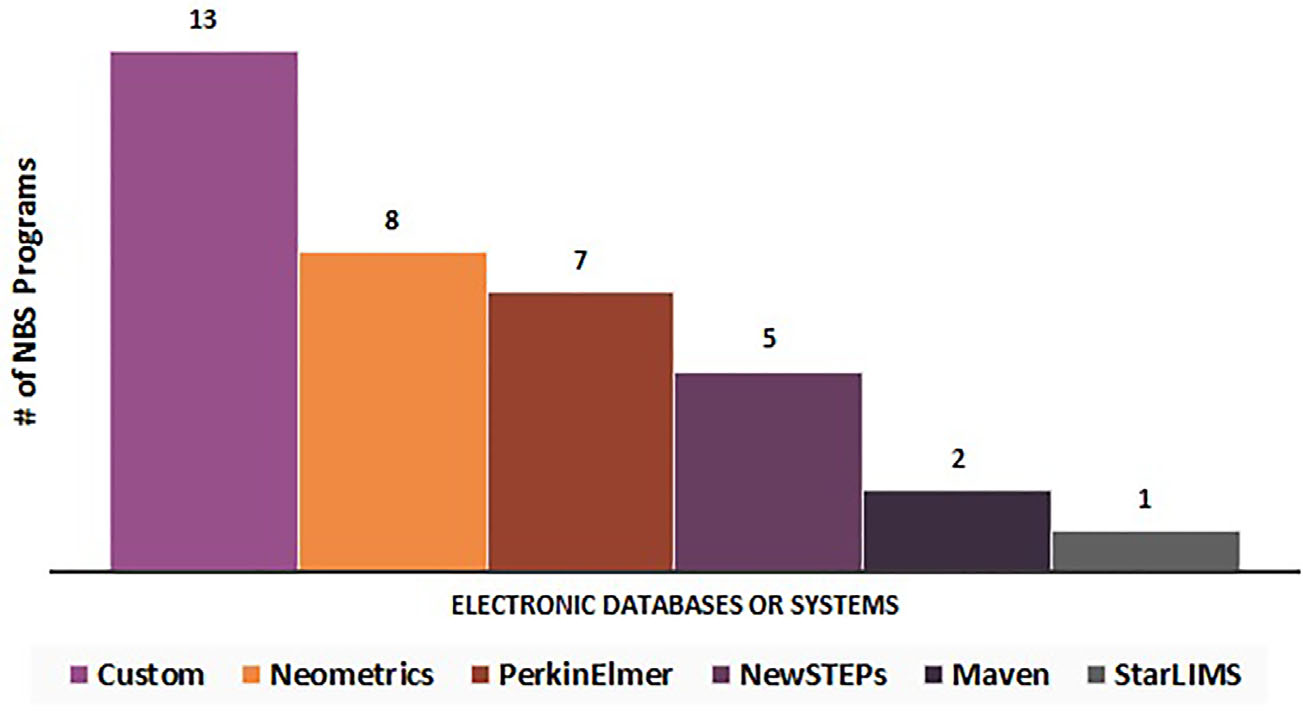
Electronic databases or systems utilized by newborn screening programs for long-term follow-up data elements (N = 33).

Respondents were asked to write in the name(s) of which electronic database/system they used. The electronic databases/systems reported can be stratified into six categories (N = 33): custom (n = 13), Neometrics (n = 8), PerkinElmer (n = 7), NewSTEPs (n = 5), Maven (n = 2), and StarLIMS (n = 1) (**Figure 6**). Fifteen percent of these NBS programs (n = 5/33) noted the utilization of multiple electronic databases or systems for data collection.

## Discussion

NBS identifies the risk for SCID in newborns by quantifying TRECs, which are produced during the normal development of T-cells. Low TREC levels facilitate the identification of patients with SCID and other serious medical conditions associated with low T-cell numbers (12). To detect TREC levels, state NBS programs have the option of utilizing a vendor kit based on end-point PCR, or a laboratory developed test (LDT) performing real-time PCR (6).

There are various benefits and limitations to both methods. The vendor kit provides ready quality assurance and quality control support; however, it was not made available to NBS programs until it was approved by the U.S. Food and Drug Administration (FDA) in December of 2014 (10).

Alternatively, LDTs can be more cost-effective and faster to implement. Many programs chose to adapt an automated *in situ* dried blood spot real time PCR TREC assay developed by the Centers for Disease Control and Prevention (CDC). This approach is limited to specific instrumentation (real-time PCR instruments with 96-well format) (6).

LDTs also offer screening flexibility when considering the addition of new conditions such as SMA. With 90% of NBS programs (n = 45/50) reporting they used LDTs, this flexibility may prove to be useful as they consider adding SMA to their state NBS panels. However, some NBS programs may also choose not to multiplex due to their individual program’s needs (6).

To date, each of these screening methods have been successfully implemented; therefore, NBS programs select the platform best suited for their laboratory’s conditions (6). An up-to-date list of screening methodologies is maintained in a publicly available Screening Methodologies and Targets Report in the NewSTEPs Data Repository (7).

To maximize the benefits of NBS, key stakeholders must be informed and families must have access to effective counseling and appropriate care over time (13). Currently, wide variation exists in communication pathways for out-of-range SCID NBS. While PCPs, other providers and immunologists are commonly notified by NBS programs of out-of-range SCID screens, less than half of NBS programs reported sharing results with hospitals or other submitters and public health nurses. Hospital staff and public health nurses may not be the medical professionals who directly share results with families; however, these stakeholders may benefit from being informed of out-of-range results. By closing the feedback loop, all stakeholders who are involved in the NBS system can be reminded of the important role they play in the early detection of disorders.

Sixteen percent of NBS programs reported they notified only one stakeholder of an out-of-range newborn screen. These survey results suggest that there are opportunities to expand notification of results to multiple stakeholders and to perform education to ensure patients are successfully connected with ongoing care.

The introduction of a new disorder on an NBS panel requires coordinated efforts to educate providers, parents, and the general public about the disorder. Examples of educational materials for these groups can include webinars, brochures, factsheets, newsletters, videos, presentations, conference exhibits, and organized awareness weeks. Many NBS programs utilize state websites as a modality for distribution of educational materials and some distribute materials by harnessing partner organizations’ networks (6).

The ACHDNC states the principal goal of long-term follow-up is to assure the best possible outcome for individuals with disorders identified through NBS (14). Long-term follow-up allows for a better understanding of the health outcomes of newborns diagnosed with SCID through NBS. This, in turn, allows for continuous quality improvement within the system to identify areas in which additional resources are needed, and to ensure infants identified with SCID by NBS are able to benefit from this early diagnosis throughout their lifespan. Unfortunately, lack of coordinated efforts, along with limited funding, has made it challenging for NBS programs to develop comprehensive long-term follow-up systems (14).

Although confirmed screening positive children are under clinical care, survey results show that less than half of all NBS programs are following patients after they receive a confirmed SCID diagnosis. Despite not having official long-term follow-up systems in place, many NBS programs are collecting data to track the number of patients lost to follow-up, track clinical outcomes of patients, evaluate the performance of specialty providers, as well as to ensure timely follow-up and treatment initiation, to track the number of out-of-range, inconclusive, false positive screens, false negative screens, and associated diagnoses, and to use in broader publication reporting.

This survey reflects a snapshot in time; completed surveys were received between November 2019 and January 2020. Thus, NBS programs who reported “not at this time” to survey questions may have been pursuing activities to get them closer to implementing screening, data exchange, more robust follow-up, strengthening educational programs, and establishing communication pathways. While this survey does reflect nuances that were reported in the “other” category, there may have been NBS programs that chose not provide additional relevant information as well.

No conclusions can be drawn as to which NBS protocols may be considered a best practice in regards to improving outcomes for newborns with out-of-range SCID screening results. Additional studies are needed to evaluate the effectiveness of stakeholder communication, educational outreach, and utility of long-term follow-up data.

## Conclusion

NBS is a comprehensive system that includes laboratory testing, diagnosis, follow-up, treatment, education and evaluation. To be effective and successful, the NBS system requires a robust laboratory capability to perform early and accurate detection of disorders, adequate resources to perform education and training in NBS, and to refer newborns for treatment upon identification of disorder.

This paper reveals opportunities to enhance the SCID NBS system by expanding communication pathways and educational outreach and establishing more formal long-term follow-up. As SCID NBS programs and treatments evolve, continued knowledge dissemination regarding lessons learned will further enable programs like SCID Compass to enhance linkages between families and services and to further elaborate long-term follow-up strategies for infants identified through NBS (15).

## Data Availability Statement

The datasets presented in this article are not readily available because access requires permission from state and territorial newborn screening programs. Requests to access the datasets should be directed to ruthanne.sheller@aphl.org.

## Author Contributions

Conceptualization: RS, SS, JO, EG, SE, CY, and TP, Methodology: RS, SS, JO, and EG. Formal analysis: RS, SS, EG, SE, CY, and TP. Writing: RS and SS. Writing—review and editing: RS, SS, EG, SE, CY, TP, JO, JB, and BF. Visualization: EG. Project administration: AH. Funding acquisition: JO. Supervision: SS and JO. All authors contributed to the article and approved the submitted version.

## Funding

The development of this Request for Proposals (RFP) application is supported by the Health Resources and Services Administration (HRSA) of the US Department of Health and Human Services (HHS) as part of an award totaling $2.97 million with 0% percentage financed with nongovernmental sources. The contents are those of the author(s) and do not necessarily represent the official views of, nor an endorsement, by HRSA, HHS or the US Government.

## Conflict of Interest

The authors declare that the research was conducted in the absence of any commercial or financial relationships that could be construed as a potential conflict of interest.
