# Supplementary material for: The Landscape of Severe Combined Immunodeficiency Newborn Screening in the United States in 2020: A Review of Screening Methodologies and Targets, Communication Pathways, and Long-Term Follow-Up Practices
Source: Front Immunol. 2020 Oct 28;11:577853. doi: 10.3389/fimmu.2020.577853 (PMC7655545; doi:10.3389/fimmu.2020.577853)
Supplement: Supplementary file 1 [file DataSheet_1.pdf]

Severe Combined Immunodeficiency (SCID) Newborn Screening Survey

Introduction:

The Association of Public Health Laboratories (APHL), in partnership with the Immune Deficiency Foundation (IDF), is interested in better understanding the landscape of severe combined immunodeficiency (SCID) newborn screening in the United States. We have developed the following survey to assess newborn screening programs' laboratory methodologies and targets, protocols for stakeholder notifications, and long-term follow-up practices as they pertain to SCID.
Specific states will not be identified in any presentations or publications as a result of this survey unless specific permission is granted. The survey should take no more than 10-15 minutes.

Instructions:

The name of your state is essential to summarizing the results. The name of the person completing the survey, their role, and contact information would be very helpful; however, these are optional. If there is other information you want to share with us that is not addressed by one of these questions, please include it in the comments section. We sincerely appreciate your time and effort in completing this survey.

Thank you.

This project is supported by the Health Resources and Services Administration (HRSA) of the U.S. Department of Health and Human Services (HHS) as part of an award totaling $4 million with 0% financed with nongovernmental sources. The contents are those of the author(s) and do not necessarily represent the official views of, nor an endorsement, by HRSA, HHS or the U.S. Government.

Demographics

1. State/Territory

▼ Select (54)

2. Please provide the below information.

- ◯ Name ____________________
- ◯ Title ____________________
- ◯ Email Address ____________________
- ◯ Phone Number ____________________

Screening Methodologies and Targets

---

3. Please review your newborn screening program's information in the table linked here. The data provided in this table was pulled directly from the NewSTEPs data repository. If you wish to make any edits or updates, please select, "Yes".

- ◯ Yes
- ◯ No

If answered No, please skip to Question 6.

---

4. What method do you use for **first-tier screening**?

- ◯ Real-time PCR
- ◯ EnLite (PerkinElmer)
- ◯ Sequencing
- ◯ Other (Please explain) ____

4a. What is this method's target for first-tier screening?

- ◯ TREC
- ◯ Other (Please explain) ____

---

5. What method do you use for **second-tier screening**?

- ◯ Real-time PCR
- ◯ EnLite (PerkinElmer)
- ◯ Sequencing
- ◯ Other (Please explain) ____________________

5a. What is this method's target for second-tier screening?

- ◯ TREC
- ◯ Other (Please explain) ____________________

---

6. Do you multiplex with Spinal Muscular Atrophy (SMA)?

- ◯ Yes
- ◯ No
- ◯ Other (Please explain) ____________________

T Cell Receptor Excision Circles (TREC) Analysis

7. How does your laboratory measure TREC?

- ○ Calculate TREC copy number per microliter using a standard curve
- ○ Use the Cq (or Ct) measure obtained directly from real time PCR instrument
- ○ Other (Please explain) ____

8. What does your laboratory use as the screening variable for SCID (i.e. the variable or metric to establish the screening cutoff for SCID)?

- ○ The calculated TREC copy number obtained for a patient.
- ○ The Cq (or Ct) measure for a patient obtained directly from the real time PCR instrument.
- ○ The Multiples of the Median (MoM) of the calculated TREC copy number obtained for a patient.
- ○ The Multiples of the Median (MoM) of the Cq (or Ct) measure obtained for a patient.
- ○ Other (Please explain) ____

9. What is your laboratory cutoff, for the screening variable used by your Program, to define “Positive”?

____

10. What is the median Cq (or Ct) for your population (please respond regardless if your lab uses Cq (or Ct) or TREC copy number as the TREC measure)?

____

11. If your laboratory calculates a TREC copy number, what is the median TREC copy number for your population?

____

Communication Pathways

12. Whom do you inform about a positive newborn screen for SCID?

- [ ] Primary care physican/ other provider
- [ ] Immunology consultant
- [ ] Public health nurse
- [ ] Family
- [ ] Hospital or other submitter
- [ ] Other (Please explain) ____________________

13. How are they contacted?

- [ ] Phone
- [ ] Letter
- [ ] Certified Letter
- [ ] Fax
- [ ] Email
- [ ] Text
- [ ] Other (Please explain) ____________________

14. How do you ensure information was received?

- ☐ No protocol
- ☐ Phone log
- ☐ Electronic log
- ☐ Certified letter receipt
- ☐ Return fax
- ☐ Return email
- ☐ Return text
- ☐ Other (Please explain) ____

Education

15. Do you have a targeted education plan for the following?

	Yes	No
Health Care Providers	○	○
Parents	○	○
General Public	○	○

Long-Term Follow-Up

16. Do you follow patients once they have a confirmed SCID diagnosis?

○ Yes

○ No

○ Other (Please explain) ____

16a. If Yes, through what age do you follow these patients?

____

17. Do you collect any of the following data on patients who receive an abnormal SCID newborn screen?

- ☐ Where the patient is currently being seen by a specialty provider
- ☐ Whether the patient is currently being seen by a primary care physician (PCP)
- ☐ Date of the last visit with the specialty provider
- ☐ Data of the last visit with the PCP
- ☐ Current patient treatment regimen
- ☐ Changes to treatment regimen
- ☐ Updated patient clinical data
- ☐ Patient's developmental progress
- ☐ Contact information of current PCP
- ☐ Other (Please explain) ______________________________
- ☐ N/A

18. How is your state/territory NBS program using the collected data?

- ☐ Track number of individuals lost to follow-up
- ☐ Track clinical outcomes of patients
- ☐ Evaluate the performance of specialty providers (physicians, nurses, and allied health professionals)
- ☐ Assess the needs of individuals/families for services
- ☐ Conduct research (e.g. cost-benefit analysis of testing)
- ☐ Other (Please explain) ____
- ☐ N/A

---

19. Is the collected data entered into an Electronic System or Database?

- ○ Yes
- ○ No

19a. If Yes, what Electronic System or Database do you use?

____

End of Block: Default Question Block

Start of Block: Block 1

Survey feedback questions

---

20. To help APHL improve their survey development, please rate the below statements based on your experience.

	Strongly Disagree (1)	Disagree (2)	Agree (3)	Strongly Agree (4)
The survey questions were clear and understandable.	○	○	○	○
The length of time to complete the survey was reasonable.	○	○	○	○

---

21. How can this survey be improved?

____

____

____

____

____

End of Block: Block 1
