# Supplementary figures and images for: The Landscape of Severe Combined Immunodeficiency Newborn Screening in the United States in 2020: A Review of Screening Methodologies and Targets, Communication Pathways, and Long-Term Follow-Up Practices
Source: Front Immunol. 2020 Oct 28;11:577853. doi: 10.3389/fimmu.2020.577853 (PMC7655545; doi:10.3389/fimmu.2020.577853)

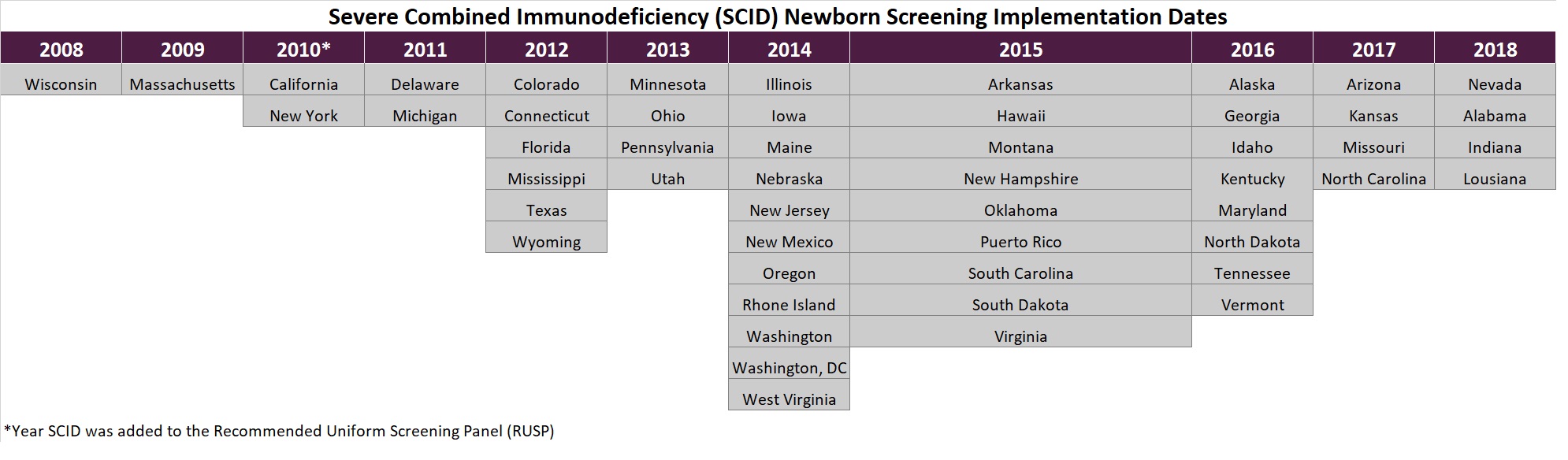

Supplement: Supplementary file 2 [file Image_1.jpg]
